# Structural and Dynamic Elucidation of a Non-acid PPAR*γ* Partial Agonist: SR1988

**DOI:** 10.11131/2018/101350

**Published:** 2018

**Authors:** Rebecca L. Frkic, Benjamin S. Chua, Youseung Shin, Bruce D. Pascal, Scott J. Novick, Theodore M. Kamenecka, Patrick R. Griffin, John B. Bruning

**Affiliations:** 1 Institute for Photonics and Advanced Sensing (IPAS), School of Biological Sciences, The University of Adelaide, Adelaide, South Australia 5005, Australia; 2 Department of Molecular Medicine, The Scripps Research Institute, Jupiter, Florida 33458, United States; 3 Omics Informatics LLC, 1050 Bishop Street #517, Honolulu, HI, 96813, Hawaii

**Keywords:** PPAR*γ*, partial agonist, nuclear receptor, type 2 diabetes, HDX

## Abstract

Targeting peroxisome proliferator-activated receptor *γ* (PPAR*γ*) by synthetic compounds has been shown to elicit insulin sensitising properties in type 2 diabetics. Treatment with a class of these compounds, the thiazolidinediones (TZDs), has shown adverse side effects such as weight gain, fluid retention, and congestive heart failure. This is due to their full agonist properties on the receptor, where a number of genes are upregulated beyond normal physiological levels. Lessened transactivation of PPAR*γ* by partial agonists has proved beneficial in terms of reducing side effects, while still maintaining insulin sensitising properties. However, some partial agonists have been associated with unfavourable pharmacokinetic profiles due to their acidic moieties, often causing partitioning to the liver. Here we present SR1988, a new partial agonist with favourable non-acid chemical properties. We used a combination of X-ray crystallography and hydrogen/deuterium exchange (HDX) to elucidate the structural basis for reduced activation of PPAR*γ* by SR1988. This structural analysis reveals a mechanism that decreases stabilisation of the AF2 coactivator binding surface by the ligand.

## 1. Introduction

PPAR*γ* is a ligand-modulated transcription factor belonging to the nuclear receptor superfamily. It forms a heterodimer with retinoid X receptor *α* (RXR*α*) to play key roles in metabolism and the maintenance of glucose homeostasis through modulating numerous target genes [1,2]. The receptor is a well-established target for treatments of type 2 diabetes (T2DM) for its roles in modulating insulin sensitivity in peripheral tissues.

Sequence analysis and crystallographic studies reveal that PPAR*γ* displays the conserved nuclear receptor domain architecture, comprising of the activation function 1 domain essential for ligand-independent coregulator binding, a DNA binding domain (DBD), a hinge region, and a ligand binding domain (LBD). The LBD binds a number of endogenous ligands, as well as facilitating dimerisation with RXR*α*, and contains a regulatory activation function 2 (AF2) region which binds coregulators in a ligand-dependant manner [3]. Crystal structures of the LBD show that the domain contains 13 *α*-helices (H1-12 and 2’) and a small *β*-sheet, conforming to the canonical nuclear receptor LBD tertiary fold. The ligand binding pocket within the LBD is lined with hydrophobic residues, enabling binding of predominantly hydrophobic endogenous ligands.

The thiazolidinediones (TZDs) are a class of full agonist modulators of PPAR*γ*, which include rosiglitazone (Avandia, GSK) and pioglitazone (Actos, Takeda). Crystal structures of rosiglitazone-bound PPAR*γ* LBD have shown that the thiazolidinedione head group forms robust stabilising interactions with the activation helix (HI2) to enable coactivator binding and initiation of target gene expression [3–7]. Rosiglitazone and pioglitazone have previously been prescribed to treat type 2 diabetes, but their use has been hampered by harmful side effects associated with supraphysiological activation of PPAR*γ* target genes. The upregulation of these genes leads to weight gain, loss of bone density, renal fluid retention and plasma volume expansion, oedema, and heart failure. These side effects have led to the clinical use of TZDs being restricted in most cases and highlights the necessity for improved antidiabetic agents targeting PPAR*γ*.

In light of the issues with TZDs as antidiabetics, research has focused on developing ligands of PPAR*γ* with reduced activation of the receptor which show a more favourable side effect profile due to a reduced expression of PPAR*γ* target genes. Although these ligands show low activation of the receptor, they still exhibit robust antidiabetic effects comparable to TZDs. It has been established that the insulin sensitising properties of ligands of PPAR*γ* are attributed to their ability to block phosphorylation of PPAR*γ* at Ser273 by extracellular signal-regulated kinases (e.g., CDK5, ERK) [8,9]. The blocking of phosphorylation is independent of the level of transactivation of the receptor induced by the ligand. It is paramount that T2DM research focusses on ligands of PPAR*γ* that have minimal activation of the receptor, such as partial agonists and antagonists, while still maintaining phosphorylation-blocking abilities.

A number of partial agonists have been investigated for their suitability as antidiabetic agents [10–15]. This approach is promising, with studies showing that the insulin-sensitising properties remain in these compounds with reduced transactivation of PPAR*γ*. An example of a well-studied partial agonist of PPAR*γ* is INT131, which shows effective blocking of Ser273 phosphorylation *in vitro*, indicative of its effectiveness as an antidiabetic agent, and showed lowered side effects as a result of limited transactivation of PPAR*γ* [12].

The array of endogenous and synthetic ligands capable of binding in the ligand binding pocket of PPAR*γ* have a diverse range of chemical properties [16]. These include sulphonamides, indoles, acids, benzimidazoles, thiazolidinediones, and cercosporamide derivatives [2]. The majority of these contain acid groups for stabilising helix 12 or the *β*-sheet of the LBD to mediate high affinity binding to the receptor [13]. However, acid moieties are disadvantageous in that they exhibit unfavourable pharmacokinetic profiles, with the compounds partitioning to the liver *in vivo* [14]. This could limit their effectiveness at their intended site of action; the peripheral tissues where the response to insulin occurs. Developing a non-acid ligand of PPAR*γ* with limited transactivation of the receptor will be ideal for an effective antidiabetic agent with favourable pharmacokinetics and side effect profile.

We have previously reported our findings on a ligand of PPAR*γ* which fits these criteria, SR2067, a highly potent non-acid partial agonist which displayed reduced transactivation of the receptor in a GAL4 transactivation assay [13]. Here we present an analogue of SR2067: SR1988, a non-acid partial agonist of PPAR*γ*. A transactivation assay was performed to determine the transcriptional potency of SR1988, a crystal structure of the ligand in complex with the PPAR*γ* LBD has been solved, and HDX was performed to further elucidate its mechanism of action through protein conformational dynamics.

## 2. Materials and Methods

### 2.1. Synthesis of SR1988

1-(2,4-difluorobenzyl)-2,3-dimethyl-N-(1-phenylpropyl)-1H-indole-5-carboxamide.

Step 1: NaH (1.1 equiv) was added to a solution of ethyl 2,3-dimethy 1–1 H-indole-5-carboxylate in DMF at room temperature. After 30 nnn, 2,4-difluorobenzyl bromide (1.1 equiv) was added to the reaction mixture and stirred for 1 h. After the reaction was completed, the solvent was removed in vacuo to obtain a crude residue which was purified by flash chromatography on silica gel (ethyl acetate/hexanes 10–100%) to obtain ethyl 1-(2,4-difluorobenzyl)-2,3-dimethyl-1H-indole-5-carboxylate. LC-MS 344 (M+H).

Step 2: A mixture of above compound and NaOH (10 equiv) in EtOH was refluxed at 100°C. for 2 h. The reaction mixture was cooled to room temperature, then acidified to pH-4 with saturated citric acid. The mixture was evaporated in vacuo to obtain the crude product, which was precipitated in water and Altered to obtain 1-(2,4-difluorobenzyl)-2,3-dimethyl-1H-indole-5-carboxylic acid which was used without further purification. LC-MS 316 (M+H).

Step 3: To a mixture of this acid in DMF was added DIPEA (1.3 equiv) and HATU (1.2 equiv). The mixture was stirred for 5 min, and then *α*-ethylbenzylamine (1.1 equiv) was added. The reaction mixture was stirred at room temperature for 1 h. After the reaction was completed, the solvent was removed in vacuo to obtain the crude which was purified by flash chromatography on silica gel (ethyl acetate/hexanes 10–100%) to obtain 1-(2,4-difluorobenzyl)-2,3-dimethyl-N-(1-phenylpropyl)-1H-indole-5-carboxamide as a colourless solid. LC-MS 433 (M+H).

### 2.2. Synthesis of SR2067

1-(naphthalen-1-ylsulfonyl)-N-(1-phenylpropyl)-1H-indole-5-carboxamide.

Step 1: To a mixture of 1 H-indole-5-carboxylic acid in DMF was added DIPEA (1.3 equiv) and HATU (1.2 equiv). The mixture was stirred for 5 min, and then *α*-ethylbenzylamine (1.1 equiv) was added. The reaction mixture was stirred at room temperature for 1 h. After the reaction was completed, the solvent was removed in vacuo to obtain the crude which was purified by flash chromatography on silica gel (ethyl acetate/hexanes 10–100%) to obtain N-(1-phenylpropyl)-1H-indole-5-carboxamide as a colourless solid.

Step 2: 1-naphthylsulfonyl chloride (1.1 equiv) and benzyltriethylammonium chloride (0.5 equiv) were added to a solution of N-(1-phenylpropyl)-1H-indole-5-carboxamide in CH_2_Cl_2_ and KOH (1.3 equiv) at room temperature. After the reaction was judged complete by analytical reverse-phase HPLC analysis, the solvent was evaporated and the crude residue was purified by silica gel column chromatography (ethyl acetate/hexanes 10–100%) to afford 1-(naphthalen-1-ylsulfonyl)-N-(1-phenylpropyl)-1H-indole-5-carboxamide as a colourless solid. LC-MS 469 (M+H).

### 2.3. Cell-based transactivation assay

HEK293T cells (ATCC; cat# CRL-3216) were cotransfected in batch by adding 4.5 *μ*g human GAL4-PPAR*γ*-Hinge-LBD, with 4.5 *μ*g 5X multimerized UAS-luciferase reporter and 27 *μ*L X-treme Gene 9 transfection reagent in serum-free Opti-mem reduced serum media (Gibco). After 18-h incubation at 37 °C in a 5% CO_2_ incubator, transfected cells were plated in quadruplicate in white 384-well plates (Perkin Elmer) at a density of 10,000 cells per well. After replating, cells were treated with either DMSO only or the indicated compounds in increasing doses from 2 pM–10 *μ*M. After 18-h incubation, treated cells were developed with Brite Lite Plus (Perkin Elmer) and read in 384-well Luminescence Perkin Elmer EnVision Multilabel plate reader. Graphs were plotted as fold change of treated cells over DMSO-treated control cells.

### 2.4. Protein purification, complex formation and crystallisation

6xHis-hPPAR*γ* LBD (res 205–477) was expressed using a pET-1 la expression vector in BL21DE3 *E. coli* cells. Expression was induced at mid-log phase using 0.5mM IPTG at 16°C overnight. Cells were harvested and lysed using a cell disruptor in 20mM Tris 8.0, 0.5M NaCl, 10mM imidazole, and 2mM BME. Protein was purified using 5mL Ni^2+^ IMAC FF Crude Column (GE Healthcare), followed by size exclusion chromatography using a HiPrep 26/60 Sephacryl S 300 HR column. The protein was concentrated to 10mg/mL using a centrifugal concentrator with a 10,000 molecular weight cut-off. Crystals were formed at 16°C using the hanging drop method. The well consisted of 1.2M sodium citrate and 0.1M Tris 8.0. The hanging drop was formed by mixing 1 *μ*L of well solution with 1 *μ*L of apo protein solution.

### 2.5. Structure determination

Apo crystals of PPAR*γ* were soaked with SR1988. SR1988 was soaked at a concentration of 2.5mM for 3 weeks at 16 °C. 15% ethylene glycol was used as a cryoprotectant. Diffraction data were collected at the MX1 beamline at the Australian Synchrotron. 360° of diffraction data at 1 degree rotations were collected for the PPAR*γ* + SR1988 crystal at one second exposure times with 85% attenuation of the beam. Processing of the data was performed using iMosflm (CCP4), which processed reflections from 58–2.4Å with space group C 1 2 1. Molecular replacement was performed using PhaserMR with PDB 4R06 (with water molecules and ligands removed) as the search model. Eight rounds of refinement in Phenix.refine showed that there was significant density for SR1988 in the binding pocket of chain A. Manual rebuilding in Coot followed by refinement in Phenix was carried out until R-factors converged. Molprobity was used for structure validation.

### 2.6. Hydrogen/deuterium exchange (HDX) detected by mass spectrometry (MS)

Differential HDX-MS experiments were conducted as previously described with some modifications [17].

#### 2.6.1. Peptide identification

Peptides were identified using tandem MS (MS/MS) with an Orbitrap mass spectrometer (Q Exactive, Thermo Fisher). Product ion spectra were acquired in data-dependent mode with the top five most abundant ions selected for the product ion analysis per scan event. The MS/MS data files were submitted to Mascot (Matrix Science) for peptide identification. Peptides included in the HDX analysis peptide set had a MASCOT score greater than 20 and the MS/MS spectra were verified by manual inspection. The MASCOT search was repeated against a decoy (reverse) sequence and ambiguous identifications were ruled out and not included in the HDX peptide set.

#### 2.6.2. HDX-MS analysis

Protein (10 *μ*M) was incubated with the respective ligands at a 1:10 protein-to-ligand molar ratio for 1 h at room temperature. Next, 5 *μ*l of sample was diluted into 20 *μ*l D_2_O buffer (20 mM Tris-HCl, pH 7.4; 150 mM NaCl; 2 mM DTT) and incubated for various time points (0, 10, 60, 300, and 900 s) at 4°C. The deuterium exchange was then slowed by mixing with 25 *μ*l of cold (4°C) 3 M urea and 1% trifluoroacetic acid. Quenched samples were immediately injected into the HDX platform. Upon injection, samples were passed through an immobilized pepsin column (2mm × 2cm) at 200 *μ*l min^−1^ and the digested peptides were captured on a 2mm × 1cm C_8_ trap column (Agilent) and desalted. Peptides were separated across a 2.1mm × 5cm C_18_ column (1.9 *μ*l Hypersil Gold, Thermo Fisher) with a linear gradient of 4% - 40% CH_3_CN and 0.3% formic acid, over 5 min. Sample handling, protein digestion and peptide separation were conducted at 4°C. Mass spectrometric data were acquired using an Orbitrap mass spectrometer (Q Exactive, Thermo Fisher). HDX analyses were performed in triplicate, with single preparations of each protein ligand complex. The intensity weighted mean m/z centroid value of each peptide envelope was calculated and subsequently converted into a percentage of deuterium incorporation. This was accomplished by determining the observed averages of the undeuterated and fully deuterated spectra and using the conventional formula described elsewhere [18]. Statistical significance for the differential HDX data is determined by an unpaired t-test for each time point, a procedure that is integrated into the HDX Workbench software [19]. Corrections for back-exchange were made on the basis of an estimated 70% deuterium recovery, and accounting for the known 80% deuterium content of the deuterium exchange buffer.

## 3. Results and Discussion

### 3.1. Design and transcriptional activity of non-acid PPAR*γ* ligand SR1988

The pharmacokinetic liabilities of PPAR*γ* ligands with acid moieties has prompted the investigation of ligands designed to bind PPAR*γ* with high affinity without acidic properties. Our previously reported non-acid partial agonist SR2067 displayed reduced transactivation of PPAR*γ* while still maintaining high potency binding to the receptor [13]. We sought to probe the effects of modifying SR2067 from the site of the 1-(methylsulphonyl)naphthalene region of the ligand by generating SR1988, which still maintained the desired non-acid characteristics of SR2067.

**Figure 1:**
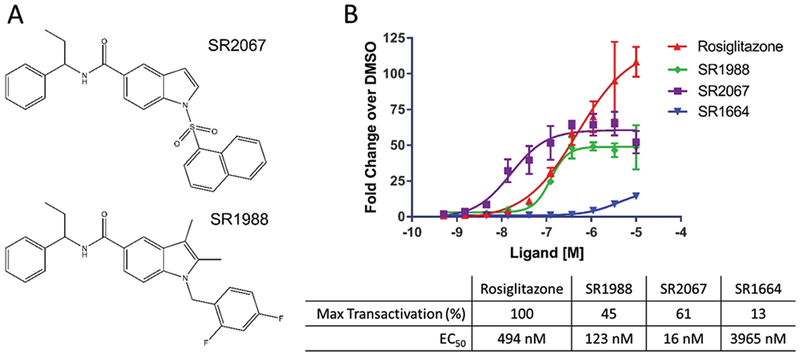
SR1988 is a non-acid partial agonist of PPAR*γ*. (A) Chemical structure of SR2067 and its analogue SR1988. (B) Transactivation data for SR1988. The transcriptional activity of SR1988 on PPAR*γ* was determined using a GAL4 transactivation assay. Data points were repeated in triplicate. Maximum transactivation values are normalised to Rosiglitazone.

SR1988 contains a central 2, 3-dimethylindole moiety flanked by a 2, 4-difluorobenzene and a hydrophobic phenylpropyl group with a terminal ethyl moiety joined by an amide linker (Figure 1A). The presence of key hydrophobic moieties within the ligand is vital for binding to the hydrophobic ligand binding pocket of PPAR*γ*.

Non-acid agonists of PPAR*γ* are advantageous in that they have ideal pharmacokinetic attributes in terms of preventing partitioning to the liver, and can carry out their insulin-sensitising properties in the required organs [13]. It is for this reason the non-acid properties of SR1988 makes it an improvement on previous partial agonists, while still retaining the advantages attributed to partial agonists as antidiabetic agents.

We have previously reported that SR2067 displays a maximal transactivation approximately 55-fold higher than DMSO, 60% relative to the maximal transactivation of rosiglitazone [13]. We performed a cell-based transactivation assay in order to compare the level of PPAR*γ* activation exhibited by SR1988 (Figure 1B). This revealed a maximal transactivation by SR1988 approximately 50-fold higher than DMSO, 45% relative to the maximal transactivation exhibited by rosiglitazone. This is typical of partial agonists and is a desired characteristic in ligands of PPAR*γ* for the treatment of type 2 diabetes. The low activation of the receptor by ligands means that PPAR*γ* target genes will not be expressed at a supraphysiological level, indicative of reduced side effects in comparison to TZDs.

The concentration of SR1988 that gave half maximal transactivation (EC_50_) was 123nM, a lower concentration than the EC_50_ of rosiglitazone, which gave half maximal transactivation at 494nM. This indicates that SR1988 is more potent than rosiglitazone in binding, and represents a major improvement on rosiglitazone in addition to the desired decrease in maximal PPAR*γ* activation. SR2067 displayed an EC_50_ value of 16nM, approximately an 8-fold lower EC_50_ than SR1988, suggesting that SR2067 is somewhat a more highly potent ligand of PPAR*γ* than SR1988. Despite this, both EC_50_ values are suitable in terms of clinical applications as they are both in the low nanomolar range. In addition, SR1988 shows an advantage over SR2067 in terms of its lower relative transactivation of PPAR*γ*, where SR1988 exhibited a 45% transactivation of PPAR*γ*, compared to 60% shown by SR2067. This lower activation will likely correspond to a more favourable side effect profile, inarguably a more important characteristic of a drug candidate.

### 3.2. X-ray crystal structure of PPAR*γ* LBD bound to SR1988

**Figure 2:**
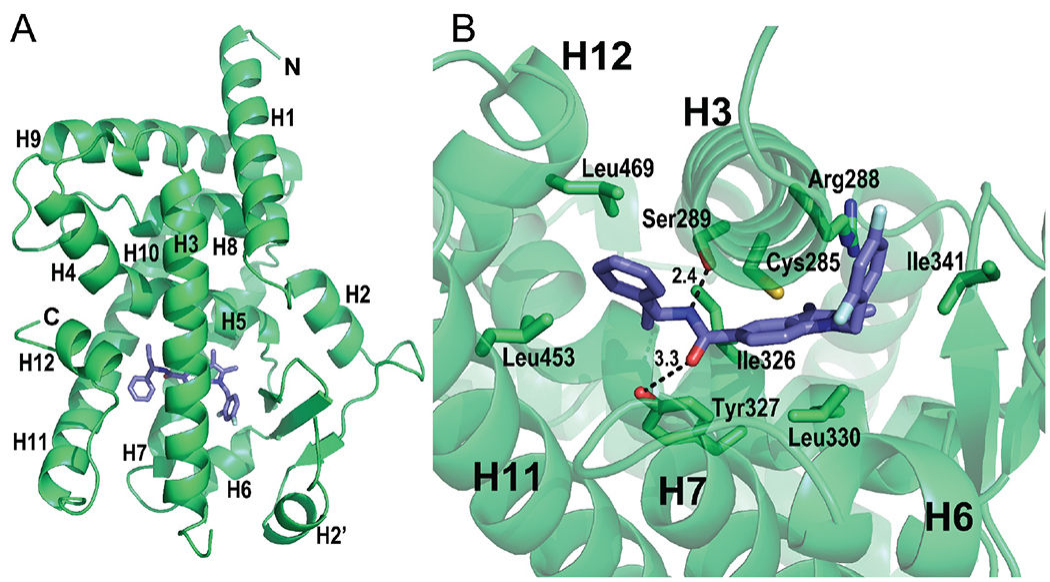
Crystal structure of PPAR*γ* LBD bound to SR1988. (A) Ribbons representation of PPAR*γ* LBD (green) bound to SR1988 (purple sticks). Only chain A is shown of the homodimeric asymmetric unit of the crystal. (B) Interactions made between SR1988 and residues lining the ligand binding pocket. Contributing side chains are shown as green sticks, with residue identities labelled.

We obtained a crystal structure of SR1988-bound PPAR*γ* LBD and solved it to 2.4Å (Figure 2A), with data refinement statistics in Table 1. The asymmetric unit of the crystal was comprised of two LBD subunits with homodimeric architecture. The protein main chain conformed to the canonical homodimeric PPAR*γ* LBD fold, with little variation in comparison to previously solved structures (RMSD of 0.58Å across 244 C*α* atoms compared with rosiglitazone-bound PPAR*γ* LBD, PDB:2PRG, and a 0.44Å RMSD over 251 C*α* atoms compared with apo PPAR*γ* LBD, PDB:1PRG). The SR1988 ligand was modelled into one chain of the homodimer, with a Polder omit map [20] displaying reduced model bias and exclusion of solvent molecules shown in Figure 1 in Supplementary Material available online at http://www.agialpress.com/journals/nurr/2018/101350/. SR1988 was bound in the ligand binding pocket of the LBD in a very similar way to a number of PPAR*γ* agonists, wrapping around H3 to make contacts with residues of H12 as well as the *β*-sheet. The central amide of the ligand forms two hydrogen bonds with the receptor (Figure 2B); the carbonyl accepting a hydrogen from Tyr327 of helix 5 over a distance of 3.3Å, and the amine donating a hydrogen to Ser289 of helix 3 in a 2.4Å hydrogen bond. In addition, hydrophobic interactions are made between the ligand and residues lining the binding pocket. The indole moiety of the ligand makes hydrophobic interactions with Cys285 (H3), Ile326 (H5) and Leu330 (H5), and the 2, 4-difluorobenzene interacts with Cys285 (H3), Arg288 (H3), and Ile341 (*β*-sheet). The phenylpropyl moiety forms hydrophobic interactions with Leu453 (H11) and Leu469 (H12).

This combination of hydrogen bonds and hydrophobic interactions made between SR1988 and PPAR*γ* are high affinity and probably entropically favourable, giving SR1988 the high potency revealed in the transactivation assay. This is key for the development of insulin-sensitising agents, as compounds that bind tightly to PPAR*γ* but have only minor transactivation of the receptor are ideal for the treatment of T2DM.

Comparison of SR1988 and SR2067 binding modes reveal almost complete similarity in the specific interactions formed with residues of the binding pocket (Figure 2 in Supplementary Material available online at http://www.agialpress.com/journals/nurr/2018/101350/), with only minor differences in hydrophobic interacting residues. The central amide forms the same hydrogen bonds as seen for SR1988, across 2.6Å for both hydrogen bonds with Tyr327 and Ser289. The unmodified phenylpropyl moiety of the two analogues are positioned in the same space within the ligand binding pocket, forming hydrophobic interactions with Leu453 (H11) and Leu469 (H12). The differing chemical structures of the ligands resulted in slight differences in their EC_50_ values, which can be attributed to minor variances in their ligand binding modes. The sulphonyl linker of SR2067 does not appear to provide an advantage in terms of favourable ligand binding compared with the carbon linker of SR1988 in the same location, as both linkers enable the ligand to orient in a way that regions of the compounds either side of the linker can form favourable interactions with the receptor. The naphthalene moiety of SR2067 occupies the same region of space in the binding pocket of PPAR*γ*, and interacts with the same residues as the 2, 4-difluorobenzene of SR1988. The higher binding potency of SR2067 could perhaps be accounted to the higher hydrophobicity of naphthalene compared with 2, 4-difluorobenzene, which would increase the likelihood of binding to PPAR*γ* and interacting with the hydrophobic pocket of the receptor. These discreet differences in the specific interactions made with the receptor by ligands of differing chemical structures highlight the importance of optimising ligands at the chemical level to enhance their potency and minimise their transactivation of PPAR*γ*.

### 3.3. Conformational dynamics of the SR1988-PPAR*γ* complex as measured by HDX

**Figure 3:**
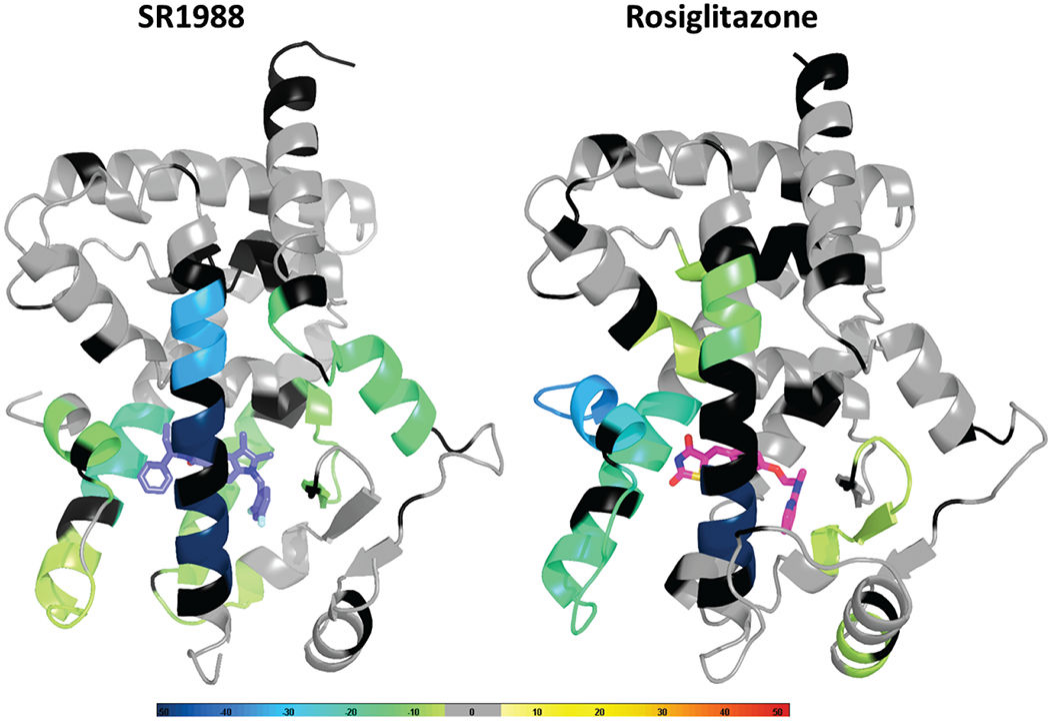
SR1988 displays a receptor stabilisation profile typical of PPAR*γ* partial agonists. HDX data for the corresponding peptides are plotted over the structures of PPAR*γ* LBD bound to rosiglitazone (PDB 2PRG) and SR1988 (PDB: 6D3E). Percentage reduction (average of all replicates and all time points) in HDX relative to apo receptor is coloured according to the key. Residues are coloured to correspond to the average percent change in deuteration between apo and SR1988 or rosiglitazone bound PPAR*γ* LBD.

In order to determine the protein dynamic stabilisation profile of the ligand-bound receptor, we performed hydrogen/deuterium exchange (HDX) on PPAR*γ* LBD in the presence of SR1988 as well as rosiglitazone (Figure 3), with HDX behaviour and peptide identities shown in Table I in Supplementary Material available online at http://www.agialpress.com/journals/nurr/2018/101350/. SR1988 displayed a HDX pattern characteristic of partial agonists. Moderate stabilisation of H12 was observed in comparison to rosiglitazone-bound PPAR*γ* LBD, where “stabilisation” refers to decreased molecular motion of H12 in its orientation relative to the remainder of the LBD. H12 of nuclear receptor LBDs has been considered a “molecular switch” for graded nuclear receptor activation, where the degree of stabilisation of H12 governs the ability for coregulators to bind, and hence determines the degree of transcriptional output exhibited by the receptor [21, 22]. Previous structural studies have shown that rosiglitazone stabilises H12 through a network of hydrogen bonds mediated by the thiazolidinedione head group of rosiglitazone, which enables robust recruitment of coactivators through stabilisation of the ligand-dependant AF2 coactivator binding surface [4]. This hydrogen bond network is lacking in SR1988, as there is a phenylpropyl group occupying this space near H12 instead of a thiazolidinedione, placing hydrophobic moieties in the pocket near the AF2. The hydrophobic moieties are not as conformationally constraining as the extensive hydrogen bond network formed between rosiglitazone and H12, which leads to a reduced stabilisation of H12 by SR1988. A decreased stabilisation of H12 suggests a lessened propensity to recruit coactivators and so will exhibit a reduced transcriptional activation of PPAR*γ*, indicating a key contributor to the mechanism of partial agonism by SR1988.

In addition, it has been shown that a graded transcriptional output of PPAR*γ* can be determined by factors extending beyond the degree of H12 stabilisation [15]. Partial agonists generally stabilise regions of H3 and the *β*-sheet of the LBD to carry out their moderate transcriptional activation [9, 15]. HDX shows that SR1988 stabilised H3 to a higher degree than rosiglitazone. This can be attributed to the hydrogen bond with Ser289 of helix 3, as well as the stabilising hydrophobic interactions with Cys285 and Arg288 identified by the crystal structure. These interactions formed between SR1988 and helix 3 of PPAR*γ* decrease the conformationally dynamic nature of the apo LBD, likely serving to allow decreased coactivator binding, giving SR1988 its partial agonist characteristics. A minor increase in *β*-sheet stabilisation was observed by HDX for SR1988 compared with rosiglitazone and DMSO controls, which has also been shown to contribute to partial agonist characteristics.

Differences in maximal transactivation efficacy of SR1988 (45%) and SR2067 (60%) can be attributed in part to differential stabilisation of regions of the LBD of PPAR*γ*. SR2067 displayed stabilising interactions with the *β*-sheet and H3 to give partial agonist properties [13]. This is similar to SR1988, although a direct comparison of the stabilisation of PPAR*γ* by SR1988 or SR2067 using HDX can provide an explanation for the differences in PPAR*γ* transactivation exhibited by SR1988 and SR2067.

## 4. Conclusion

In summary, we have used a combination of X-ray crystallography and HDX to dehne the mechanism of PPAR*γ* partial agonism by SR1988. A reduced stabilisation of H12 as well as increased stabilisation of H3 and the *β*-sheet in comparison to rosiglitazone account for moderate, partial agonist like transactivation of PPAR*γ* by SR1988. Our study demonstrates that non-acid ligands of PPAR*γ* still exhibit high affinity binding to the receptor, despite their lack of acidic moieties to stabilise regions of the LBD. These hndings are applicable to the future development of insulin sensitising agents, particularly for its low transactivation as well as nonacid characteristics, giving it more ideal pharmacokinetic properties and likely a favourable side effect profile.

## Figures and Tables

**Table 1:** Crystallographic data statistics.

PDB accession code	6D3E
Space group	C 1 2 1
Cell dimensions	
*a, b, c* (Å)	93.04, 62.51, 118.65
*α, β, γ* (°)	90, 102.32, 90
X-ray source	Australian Synchrotron MX1
Wavelength (Å)	0.9537
Resolution range (Å)	58.0-2.40 (2.49-2.40)
R_merge_ (%)^a^	0.12 (1.18)
R_pim_ (%)	0.075 (0.794)
Observations	155718 (13591)
Unique reflections	26013 (2563)
Mean (I)/σ(I)	11.8 (1.8)
Completeness	98.3 (93.0)
Multiplicity	6.0 (5.3)
CC(1/2)	0.998 (0.601)
Structure refinement	
Resolution range (Å)	39.5-2.4
R_work_^b^	0.206 (0.283)
R_free_^c^	0.264 (0.316)
Total number of	
Non-hydrogen atoms	4420
Protein atoms	4210
Ligand atoms	32
Water molecules	178
RMSD	
Bond length (Å)	0.002
Bond angle (deg)	0.42
B-factors (Å^2^)	
Overall	63.7
Average protein atoms	63.5
Average ligand atoms	105.5
Average solvent	59.4
Ramachandran statistics	
Most favoured regions (%)	96.71
Allowed regions (%)	3.10
Disallowed regions (%)	0.19

Values in parentheses correspond to the last shell.

a R_merge_ = Σ |I − <I>| / Σ I.

b R_work_ = Σ |F_*o*_−F_*c*_| / Σ |F_*o*_| for all data excluding data used to calculate R_free_.

c R_free_ = Σ |F_*o*_−F_*c*_| / Σ |F_*o*_|. for all data.

## References

[1] Issemann I, Prince RA, Tugwood JD, and Green S, “The retinoid X receptor enhances the function of the peroxisome proliferator activated receptor,” Biochimie, vol. 75, no. 3-4, pp. 251–256, 1993. 8389594 10.1016/0300-9084(93)90084-6

[2] Kroker AJ and Bruning JB, “Review of the structural and dynamic mechanisms of PPAR*γ* partial agonism,” PPAR Research, vol. 2015, Article ID 816856, 15 pages, 2015. 10.1155/2015/816856 PMC4578752 26435709

[3] Chandra V, Huang P, Hamuro Y , “Structure of the intact PPAR-*γ*-RXR-*α* nuclear receptor complex on DNA,” Nature, vol. 456, no. 7220, pp. 350–356, 2008. 19043829 10.1038/nature07413 PMC2743566

[4] Nolte RT, Wisely GB, Westin S , “Ligand binding and co-activator assembly of the peroxisome proliferator-activated receptor-*γ*,” Nature, vol. 395, no. 6698, pp. 137–143, 1998. 9744270 10.1038/25931

[5] Gampe RT Jr., Montana VG, Lambert MH , “Asymmetry in the PPAR*γ*/RXR*α* crystal structure reveals the molecular basis of heterodimerization among nuclear receptors,” Molecular Cell, vol. 5, no. 3, pp. 545–555, 2000. 10882139 10.1016/s1097-2765(00)80448-7

[6] Gelin M, Delfosse V, Allemand F , “Combining ‘dry’ co-crystallization and in situ diffraction to facilitate ligand screening by X-ray crystallography,” Acta Crystallographica Section D: Biological Crystallography, vol. 71, pp. 1777–1787, 2015. 26249358 10.1107/S1399004715010342

[7] Liberato MV, Nascimento AS, Ayers SD , “Medium chain fatty acids are selective peroxisome proliferator activated receptor (PPAR) *γ* activators and Pan-PPAR partial agonists,” PLoS ONE, vol. 7, no. 5, Article ID e36297, 2012. 10.1371/journal.pone.0036297 PMC3359336 22649490

[8] Banks AS, McAllister FE, Camporez JPG , “An ERK/Cdk5 axis controls the diabetogenic actions of PPARgamma,” Nature, vol. 517, no. 7534, pp. 391–395, 2015. 25409143 10.1038/nature13887 PMC4297557

[9] Choi JH, Banks AS, Estall JL , “Anti-diabetic drugs inhibit obesity-linked phosphorylation of PPAR*γ* 3 by Cdk5,” Nature, vol. 466, no. 7305, pp. 451–456. 2010. 20651683 10.1038/nature09291 PMC2987584

[10] Frkic RL, He Y, Rodriguez BB , “Structure-Activity Relationship of 2,4-Dichloro-N-(3,5-dichloro-4-(quinolin-3-yloxy)phenyl)benzenesulfonamide (INT131) Analogs for PPAR*γ*-Targeted Antidiabetics,” Journal of Medicinal Chemistry, vol. 60, no. 11, pp. 4584–4593, 2017. 28485590 10.1021/acs.jmedchem.6b01727 PMC5537074

[11] Taygerly JP, McGee LR, Rubenstein SM , “Discovery of INT131: a selective PPAR*γ* modulator that enhances insulin sensitivity,” Bioorganic & Medicinal Chemistry, vol. 21, no. 4, pp. 979–992, 2013. 23294830 10.1016/j.bmc.2012.11.058

[12] Motani A, Wang Z, Weiszmann J , “INT131: a selective modulator of PPAR*γ*,” Journal of Molecular Biology, vol. 386, no. 5, pp. 1301–1311, 2009. 19452630 10.1016/j.jmb.2009.01.025

[13] van Marrewijk LM, Polyak SW, Hijnen M , “SR2067 reveals a unique kinetic and structural signature for PPAR*γ* partial agonism,” ACS Chemical Biology, vol. 11, no. 1, pp. 273–283, 2016. 26579553 10.1021/acschembio.5b00580 PMC4819005

[14] Sime M, Allan AC, Chapman P , “Erratum: Discovery of GSK1997132B a novel centrally penetrant benzimidazole PPAR*γ* partial agonist (Bioorganic and Medicinal Chemistry Letters (2011) 21 (5568–5572)),” Bioorganic & Medicinal Chemistry Letters, vol. 23, no. 4, p. 1143, 2013. 10.1016/j.bmcl.2011.06.088 21798739

[15] Bruning JB, Chalmers MJ, Prasad S , “Partial agonists activate PPAR*γ* using a helix 12 independent mechanism,” Structure, vol. 15, no. 10, pp. 1258–1271, 2007. 17937915 10.1016/j.str.2007.07.014

[16] Sauer S, “Ligands for the nuclear peroxisome proliferator-activated receptor gamma,” Trends in Pharmacological Sciences, vol. 36, no. 10, pp. 688–704, 2015. 26435213 10.1016/j.tips.2015.06.010

[17] Chalmers MJ, Busby SA, Pascal BD , “Probing protein ligand interactions by automated hydrogen/deuterium exchange mass spectrometry,” Analytical Chemistry, vol. 78, no. 4, pp. 1005–1014, 2006. 16478090 10.1021/ac051294f

[18] Zhang Z and Smith DL, “Determination of amide hydrogen exchange by mass spectrometry: A new tool for protein structure elucidation,” Protein Science, vol. 2, no. 4, pp. 522–531, 1993. 8390883 10.1002/pro.5560020404 PMC2142359

[19] Pascal BD, Willis S, Lauer JL , “HDX workbench: software for the analysis of H/D exchange MS data,” Journal of The American Society for Mass Spectrometry, vol. 23, no. 9, pp. 1512–1521, 2012. 22692830 10.1007/s13361-012-0419-6 PMC3808162

[20] Liebschner D, Afonine PV, Moriarty NW , “Polder maps: Improving OMIT maps by excluding bulk solvent”, Acta Crystallographica Section D: Structural Biology, vol. 73, no. 2, pp. 148–157, 2017. 28177311 10.1107/S2059798316018210 PMC5297918

[21] Nagy L and Schwabe JWR, “Mechanism of the nuclear receptor molecular switch,” Trends in Biochemical Sciences, vol. 29, no. 6, pp. 317–324, 2004. 15276186 10.1016/j.tibs.2004.04.006

[22] Batista MRB and Martínez L, “Dynamics of nuclear receptor Helix-12 switch of transcription activation by modeling time-resolved fluorescence anisotropy decays,” Biophysical Journal, vol. 105, no. 7, pp. 1670–1680, 2013. 24094408 10.1016/j.bpj.2013.07.032 PMC3791304

